# New-Onset Cervical Lymphadenopathy in a Patient Undergoing Treatment of Pulmonary *Mycobacterium avium* Complex Infection: Toxoplasmosis Lymphadenitis

**DOI:** 10.1155/2020/8876240

**Published:** 2020-09-07

**Authors:** Chia-Yu Chiu, Amara Sarwal, Peter Yangga, Dasol Kang, Addi Feinstein

**Affiliations:** ^1^Department of Internal Medicine, Lincoln Medical Center, New York City, NY, USA; ^2^Department of Internal Medicine, Section of Infectious Diseases, Lincoln Medical Center, New York City, NY, USA

## Abstract

Immunocompetent hosts with toxoplasmosis are usually asymptomatic. However, *T. gondii* can present as an acute systemic infection. Symptomatic patients usually have a benign, self-limited course that typically lasts from a few weeks to months. Herein, we present a 66-year-old immunocompetent female who developed dysphagia and new-onset cervical lymphadenopathy during pulmonary *Mycobacterium avium complex* treatment.

## 1. Introduction

Toxoplasmosis infection in immunocompetent patients are usually asymptomatic, self-limiting, and do not require treatment. Clinical manifestations include fever, chills, and myalgia for several days. Some patients may develop generalized lymphadenopathy, and the majority develop bilateral, symmetrical, and nontender cervical adenopathy [1–3]. Treatment is initiated in patients who are pregnant, having prolonged symptoms, and having ocular involvement, pneumonitis, myocarditis, or meningoencephalitis. The *T. gondii* seroprevalence in USA-born persons between the ages of 12–49 years in 1999–2004 was 9%, and the prevalence in foreign-born women between the ages of 15–44 years in 1999–2004 was 28.1% [3]. Previous studies showed a decrease in *T. gondii* seroprevalence in the USA over time, with a prevalence of 14.4% in 1962 and 9.5% in 1989 among military recruits [3]. Risk factors for *T. gondii* infection include persons who are foreign-born, have a lower education level, live in a crowded environment, and work in soil-related occupations [4].

## 2. Case Presentation

**Figure 1:**
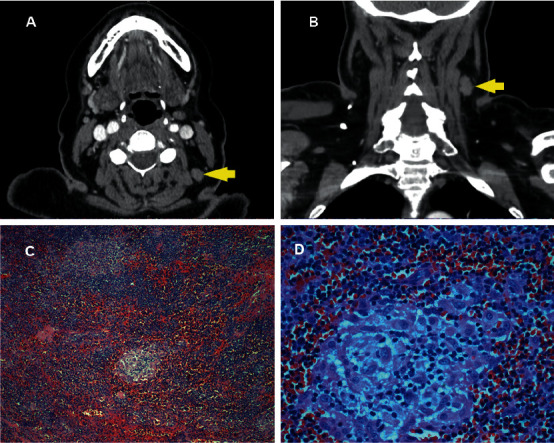
CT scan at transverse plane (a) and coronal plane (b) showing a 1.4 cm left level 5 A lymph node. No other adenopathy. No mass along the course of the aerodigestive tract. Cervical lymph node biopsy demonstrating lymph node with follicular hyperplasia, phagocytosis of nuclear debris, noncaseating epithelioid granuloma, and sinusoidal distension by plasma cells and immunoblasts (c) (x10 magnification). Higher magnification of the epithelioid granulomas (d) (x40 magnification).

A 66-year-old human immunodeficiency virus- (HIV-) seronegative female, a native of the Dominican Republic, presented to our hospital for a foreign body sensation when swallowing solid food for the past 1 month. Her medical history was notable for type 2 diabetes mellitus and pulmonary *Mycobacterium avium* complex (MAC). She was diagnosed with pulmonary MAC 15 months ago and was currently being treated with azithromycin 500 mg, rifampin 500 mg, and ethambutol 2000 mg, which was administered three times per week. Her last negative sputum culture was 1 month prior to presentation. She endorsed compliance with the medication, and her last A1C was 6.8%. She smoked 1 pack of cigarettes for the last 41 years and has abstained from smoking since the diagnosis of MAC. Her physical exam revealed a 1 cm painless, firm, and nonmobile lymph node at the left side of her neck. Laboratory test results including complete blood count, basic metabolic panel, and liver function test were unremarkable. Barium study was negative for structural abnormality, and flexible fibrotic laryngoscopy showed normal appearance of the larynx. Computed tomography (CT) showed a lymph node, 1.4 cm in size, at the level 5 of the left neck. After an additional 6 months of MAC treatment, her dysphagia did not improve. Repeat neck CT revealed the 1.4 cm lymph node, unchanged from previous (Figures 1(a) and 1(b)). Biopsies of the abnormal lymph node were taken (Figures 1(c) and 1(d)).

**Figure 2:**
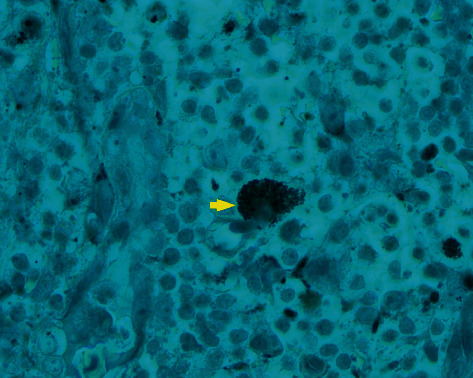
*Toxoplasma gondii* bradyzoite as demonstrated by GMS stain (x100 magnification).

The initial differential diagnostic considerations included nontuberculous mycobacterial lymphadenitis, tuberculous lymphadenitis, and bacterial, fungal, or protozoal infection. Metastatic squamous cell carcinoma arising from the aerodigestive tract or lymphoma is less likely in this setting due to lack of warning symptoms. Excisional biopsy was not performed at the beginning of presentation in this case because we decided to actively surveil while the patient was under MAC treatment, given that we successfully achieved a negative sputum culture. However, after an additional 6 months of MAC treatment with significant improvement on chest CT without a decrease in size of neck lymphadenopathy, we decided to undergo excisional biopsy of the lymph node for further evaluation. The histopathological examination revealed noncaseating epithelioid granulomas (Figure 1(d)), and Gomori methenamine silver (GMS) stain was consistent with *Toxoplasma gondii* bradyzoite (Figure 2). Serum toxoplasma IgG was 178 IU/ml, and IgM was negative. We administered trimethoprim-sulfamethoxazole (TMP-SMX) for 2 weeks.

## 3. Discussion

*T. gondii* is an obligate intracellular parasite in humans. T helper type 1 (Th1) cells are a lineage of CD4+ effector T cells that promote cell-mediated immune response and are required for host defense against intracellular bacterial and parasite pathogens. Macrophages and monocytes are responsible for restricting the proliferation of parasites. Both TNF-alpha and INF-gamma are necessary for synthesis of nitric oxide (NO) and oxygen intermediates in the cellular immune system [5]. During active tuberculosis (TB) infection, there is a decrease of Th1 activity and an increase in T helper type 2 (Th2) activity. The shift of Th1 response to Th2 response suppresses the cell-mediated immune response against toxoplasma which could reactivate latent toxoplasma or increase the susceptibility of new infection [6]. Some studies show that the prevalence of serum anti-toxoplasma IgG in TB-positive patients in Egypt, Burkina Faso, and Sudan is 100% (29/29), 52% (26/50), and 25% (7/28), respectively, which was higher than that in TB-negative patients [6–8].

In developing countries, co-infection of TB and parasitic disease has become a public concern. However, TB concomitant with toxoplasma has been understudied with a lack of review articles [9]. The earliest report found was in 1984, when a Haitian refugee presented with disseminated TB with toxoplasma encephalitis and a high suspicion for HIV [10]. The second case we found is of a four-year-old girl in Sri Lanka who had a co-infection of TB, toxoplasma, and toxocara which was confirmed by biopsy of inguinal TB lymphadenopathy, serum toxoplasma IgM, and serum toxocara IgG [11]. The third case reported was from South Korea, with an immunocompetent Indonesian worker who had disseminated TB and cerebral toxoplasmosis [12]. In developing countries, because of the high prevalence of TB and lack of diagnostic accessibility, some physicians raised the concern about a misleading diagnosis of toxoplasmosis in patients who had TB or lymphoma. TB and toxoplasmosis both are characterized by epithelioid granulomas, which could cause the two to be mistaken for each other. In addition, occasionally, toxoplasmic lymphadenitis may be confused histologically or may coexist in a lymphoma patient [1, 13, 14].

Opportunistic toxoplasmosis disease has been well discussed in HIV patients. Over the years, screening of anti-toxoplasma IgG, prophylaxis for CD4 cell count less than 100 cells/*μ*L, and highly active antiretroviral therapy have become widely used as preventative techniques. There was a correlated decline in the incidence and death of opportunistic infection of toxoplasma [3, 15]. TB/MAC and HIV co-infection also has been well recognized [15–19]. HIV increases latent TB, MAC, and toxoplasmosis reactivation by depleting CD4+ T cells and manipulating the Th1/Th2 balance. Nevertheless, the linkage between toxoplasma and TB/MAC in immunocompetent patients has not been established, although it may share a similar mechanism.

Tuberculosis-immune reconstitution inflammatory syndrome (TB-IRIS) was used to describe a collection of symptoms that occurs when patients initiate antituberculosis therapy and/or antiretroviral therapy [20–22]. TB-IRIS can present as constitutional symptoms, worsening respiratory symptoms, new radiographic abnormalities, or lymphadenopathy. The incidence of TB-IRIS is higher in HIV-infected patients following antiretroviral compared with HIV-uninfected patients, at 36% (12 of 33 patients) and 2% (1 of 55 patients), respectively [23]. The median time to onset of the TB-IRIS after initiation of anti-TB treatment is 2 weeks to 4 weeks in HIV-infected patients and 3 weeks to 8 weeks in HIV-uninfected patients [20]. TB-IRIS frequently presents as extrapulmonary manifestations (pleural or lymphadenopathy) rather than involving lung parenchyma [20, 21]. Of note, the above data are limited to TB and do not include MAC. There are sporadic case reports of MAC-IRIS, but it has been limited in HIV co-infected individuals.

To our understanding, this is the first case of toxoplasmosis concomitant with MAC in a HIV-seronegative patient. For patients who are undergoing anti-TB treatment that develop lymphadenopathy, toxoplasma reactivation should be kept in mind as a differential diagnosis.

## Data Availability

The data used to support the findings of this study are included within the article.
